# IRF1 Downregulation by Ras/MEK Is Independent of Translational Control of IRF1 mRNA

**DOI:** 10.1371/journal.pone.0160529

**Published:** 2016-08-10

**Authors:** Yumiko Komatsu, Leena Derwish, Kensuke Hirasawa

**Affiliations:** Division of BioMedical Science, Faculty of Medicine, Memorial University of Newfoundland, 300 Prince Philip Dr., St. John’s, NL, A1B 3V6, Canada; University of British Columbia, CANADA

## Abstract

Oncogenic activation of Ras/MEK downregulates the expression of interferon regulatory factor 1 (IRF1), which is a prerequisite for oncolytic viruses to replicate in cancer cells [1]. Moreover, restoration of IRF1 expression is essential to induce apoptosis of cancer cells treated with a MEK inhibitor [2]. However, the molecular mechanisms that underlie IRF1 downregulation by Ras/MEK remain unclear. In this study, we determined whether Ras/MEK activation modulates IRF1 expression at its translational level. MEK inhibition increased the activity of IRF1 promoter construct in Ras transformed NIH3T3 cells and wild type MEF, but not in IRF1 deficient MEF, indicating that IRF1 protein is required for the transcriptional activation of IRF1. By conducting reporter analysis using IRF1 5’- and 3’- UTR constructs, we determined that *cis* elements on 5’- and 3’-UTR of IRF1 mRNA are not involved in the IRF1 regulation by Ras/MEK. We further compared the recruitment of ribosomes to IRF1 mRNA in RasV12 cells treated with or without the MEK inhibitor by conducting polysome analysis. No difference was observed in the polysomal distribution of IRF1 mRNA between RasV12 cells treated with and without the MEK inhibitor. These results suggest that regulation of IRF1 translation is independent of IRF1 downregulation by Ras/MEK.

## Introduction

Oncolytic viruses preferentially replicate within cancer cells, leading to destruction of cancer cells while keeping the normal cells unharmed. Oncolytic viruses exploit tumor-specific molecular changes in cancer cells for their replication, such as p53 deficiency [3], oncogenic Ras activation [3], defects in the type I interferon (IFN)-induced antiviral response [4] and viral receptors uniquely expressed on cancer cells [5]. Our research focus has been on identifying further molecular mechanisms of viral oncolysis. We reported that IFN-sensitive oncolytic viruses can replicate in cells with constitutively active Ras (RasV12 cells) despite the presence of type I IFN [6]. Noser et al. (2007) also reported that the inhibition of Ras-Raf-MEK-ERK pathway in human cancer cell lines restored antiviral responses induced by IFN [7]. These studies clearly demonstrated that the tumor-specific molecular changes exploited by oncolytic viruses, oncogenic Ras activation and defects in the type I IFN, are connected. We further found that activated Ras/MEK suppresses the transcription of a group of IFN-inducible genes (MEK-downregulated IFN-inducible (MDII) genes) by conducting microarray analysis [8;9]. One of these MDII genes is signal transducer and activator of transcription 2 (STAT2), and its overexpression partially restores the IFN-induced antiviral response to oncolytic viruses in cells with activated Ras [10], indicating a causal relationship between Ras-mediated downregulation of MDII genes and sensitivity to oncolytic viruses. Recently, we identified interferon regulatory factor 1 (IRF1) as the transcriptional regulator of MDII genes [1;2]. Furthermore, we demonstrated that MEK inhibition restored IRF1 expression in human cancer cells and that the level of IRF1 expression defines the sensitivity of cancer cells to certain oncolytic viruses. These studies clearly demonstrate that IRF1 downregulation by Ras/MEK is the one of molecular mechanisms underlying viral oncolysis.

Ras belongs to the family of small GTPases that function as molecular switches to transduce external cellular signals to the nucleus by cycling between an inactive GDP-bound state and an active GTP-bound state [11;12]. In an active GTP-bound state, Ras recruits and activates its downstream effector Raf kinase at the plasma membrane. Activated Raf phosphorylates mitogen-activated protein kinase/ERK kinase (MEK) 1/2, which in turn phosphorylates the extracellular signal regulated kinases (ERK) 1/2. Once activated, ERKs regulate transcriptional and translational activities that control multiple cellular processes including cell growth, differentiation, proliferation, adhesion, migration, and apoptosis [13].

Nearly 30% of all human cancers have activating mutations in Ras, which varies depending on the cancer type [14]. The Ras-Raf-MEK-ERK pathway can also be stimulated by aberrant activation of its upstream signaling components of Ras, including epidermal growth factor receptor (EGFR), erb-b2 receptor tyrosine kinase 2 (HER2/neu), or SRC proto-oncogene nonreceptor tyrosine kinase (SRC) [15]. Furthermore, activating mutations of Raf are commonly found in malignant melanoma, thyroid, colorectal, and ovarian tumors [16]. Overall, the majority of cancer cells have oncogenic activation of the Ras-Raf-MEK-ERK pathway.

IRF1 is a transcription factor which regulates a number of IFN-inducible genes in response to viral infection or IFN stimulation [17–20]. IRF1 activates the transcription of critical antiviral effectors such as 2’-5’- oligoadenylate synthase (OAS), retinoic inducible gene I (RIG-I) and Viperin [21–23] and exhibits its antiviral activity against a broad range of viruses including hepatitis C virus (HCV), human immunodeficiency virus (HIV), influenza virus, vesicular stomatitis virus (VSV) and West Nile virus [22;24–28]. In addition, IRF1 is a critical regulator of immune response due to its ability to regulate expression of major histocompatibility complex (MHC) class I, transporter associated with antigen presentation 1 (TAP1), low molecular mass polypeptide 2 (LMP2) and class II transactivator (CIITA) [29–33]. Another important property of IRF1 is its anticancer role by regulating the transcription of tumor suppressors and oncogenes. While wildtype MEFs require introduction of at least two independent oncogenes to be transformed [34], Tanaka et al. (1994) demonstrated that introduction of one oncogene was sufficient to transform MEFs lacking IRF1, demonstrating the anticancer property of IRF1 [35]. The anticancer function of IRF1 is further supported by the observations that IRF1 inhibits cell transformation induced by oncogenes including c-myc, fos-B, IRF2, and EGFR [36–38]. In cancer patients, the expression level of IRF1 mRNA is negatively correlated with the tumor grade, risk of recurrence and death [39;40].

IRF1 regulates the transcription of a broad range of genes with antiviral, anticancer and immune regulatory functions [41;42]. Therefore, identifying the regulatory mechanism of IRF1 by Ras/MEK will have important implications not only in the field of oncolytic viruses but also in the field of virology, cancer biology and immunology. Here, we examined whether IRF1 expression is regulated by Ras/MEK at the translational level by conducting 5’- and 3’-UTR reporter assay and polysome analysis.

## Results

**Fig 1:**
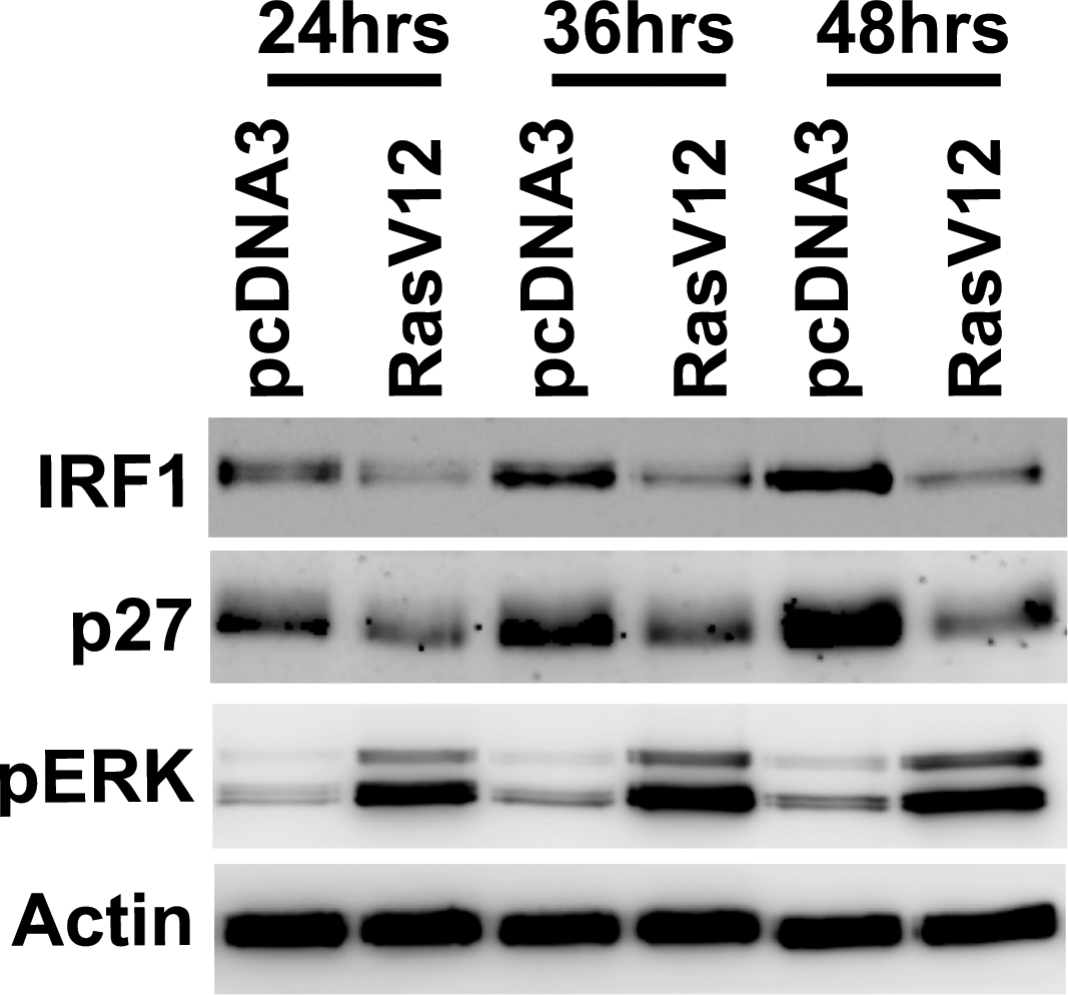
IRF1 downregulation by Ras/MEK activation. NIH3T3 cells were transfected with control pcDNA3 vector or pcDNA3 vector containing RasV12 gene. Western blot analysis was conducted to determine the expression of IRF1, p27^Kip^ and actin and the phosphorylation of ERK (pERK) at 24, 36 and 48 hours after transfection. Result shown is a representative of two independent experiments.

**Fig 2:**
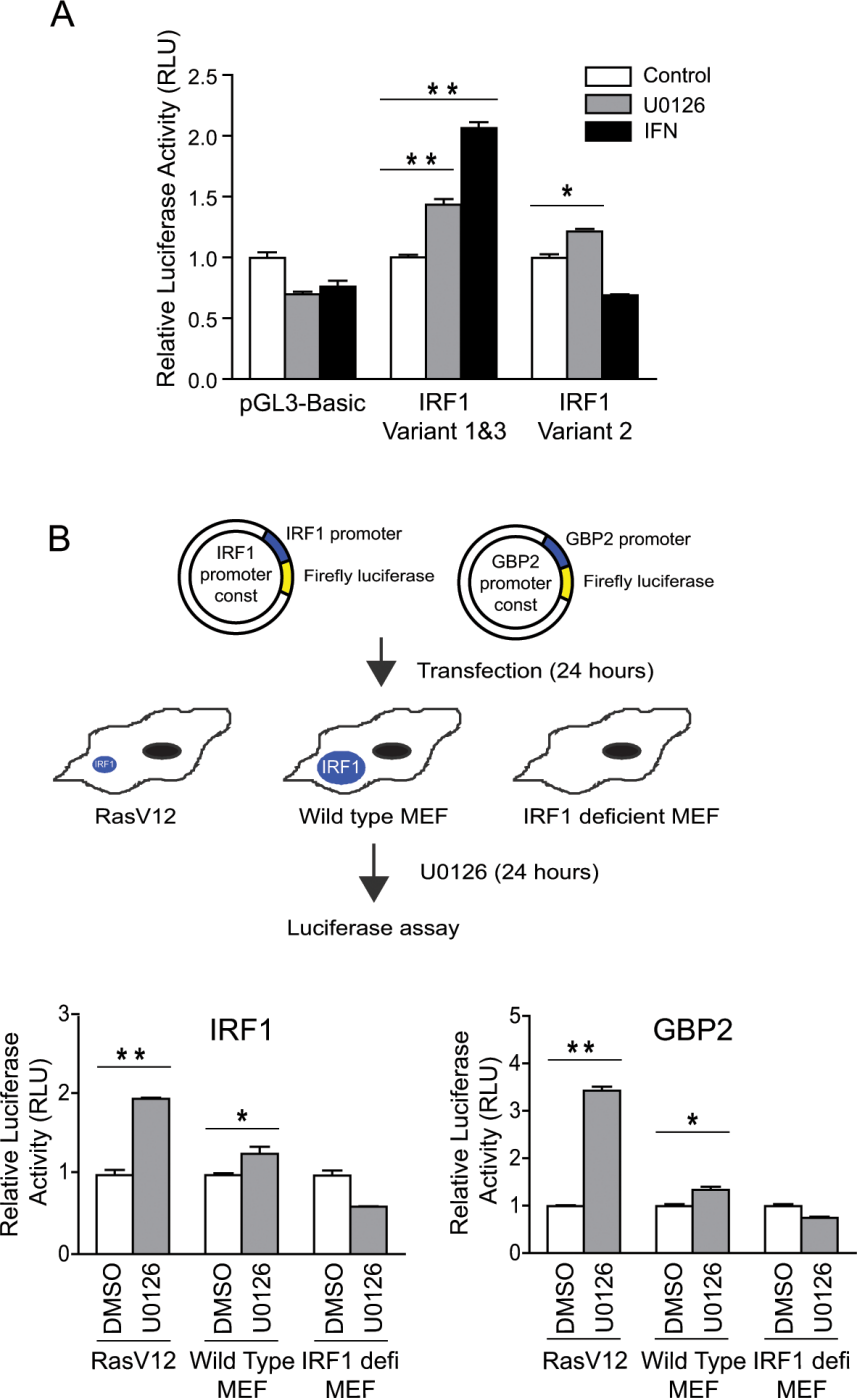
IRF1 protein is required for IRF1 promoter activity induced by MEK inhibition. (A) Control pGL3-Basic plasmid, pGL3-Basic plasmid containing promoter of IRF1 variant 1&3 or IRF1 variant 2 were transfected into RasV12 cells. At 24 hours after transfection, the cells were treated with U0126 (20μM) or IFN-α (500U/ml) for 24 hours. (B) pGL3-Basic plasmid containing IRF1 variant 1&3 promoters or GBP2 promoter was transfected into RasV12 cells, wild-type MEFs or IRF1-deficient MEFs. At 24 hours after transfection, the cells were treated with U0126 (20μM) for 24 hours. Relative luciferase activities (RLU) were reported as compared with DMSO controls (n = 3, *P<0.05, **P<0.01). Result shown is a representative of three independent experiments.

To confirm the presence of Ras/MEK and IRF1 connection in mouse fibroblast system, NIH3T3 cells were transfected with either control pcDNA3 plasmid or pcDNA3 containing RasV12 gene for 24, 36 or 48 hours (Fig 1). Increased phosphorylation of ERK indicated that the Ras/MEK pathway is activated upon RasV12 transfection. Expression of IRF1 as well as p27^Kip1^, a tumor suppressor induced by IRF1, was downregulated by RasV12 transfection, indicating that Ras/MEK activation suppresses expression and function of IRF1. In our previous study, we reported that Ras/MEK activation decreases IRF1 expression both at the protein and mRNA level [1]. As IRF1 is an auto-regulatory transcriptional activator for its own synthesis [43], IRF1 transcriptional and translational activities are closely related. To determine which IRF1 transcription or translation is the primary target of Ras/MEK, we examined whether Ras/MEK modulates IRF1 transcription in the absence of IRF1 protein (Fig 2). Three mouse IRF1 variants have been reported in the NCBI database (NM_008390.2, NM_001159396.1, NM_001159393.1), two of which (variant 1 and 3) have the same promoter region. Variant 2 has an alternative promoter region from variant 1 and 3. We first cloned IRF1 variant 1&3 or variant 2 promoter region into pGL3-Basic vectors and tested their promoter activities in RasV12 cells (Fig 2A). Promoter activities of variant 1&3, and variant 2 to a lesser extent, significantly increased by 24 hours of MEK inhibition. Treatment with IFN-α, which is a well-known transcriptional activator of IRF1 [42], significantly increased promoter activity of IRF1 variant 1&3, but not that of IRF1 variant 2, suggesting that the IRF1 variant 2 promoter is not IFN-responsive. Considering the relevance of IRF1’s antiviral property, we used the promoter of IRF1 variant 1&3 for further studies. To determine whether IRF1 mRNA expression can be promoted by MEK inhibition in the absence of IRF1 protein, we determined the promoter activity of IRF1 variant 1&3 in RasV12, wild type or IRF1 deficient MEFs following treatment with MEK inhibitor U0126 (Fig 2B). U0126 treatment significantly increased the IRF1 promoter activity in wild-type MEFs, albeit the increase was lower than in RasV12 cells. This is likely due to less basal activation of Ras/MEK in wild-type MEF compared to in RasV12. In contrast, induction of the IRF1 promoter activity by U0126 was not observed in IRF1-deficient MEFs, suggesting that the induction of IRF1 mRNA by MEK inhibition is dependent on IRF1 protein. We also examined the promoter activity of GBP2, which is one of the IFN-inducible genes regulated by IRF1. Similarly, GBP2 promoter activity was significantly increased in RasV12 than in wild-type MEFs by U0126 treatment, while U0126-induced GBP2 promoter activity was completely abrogated in IRF1-deficient cells. Together, these data indicated that the IRF1 downregulation by Ras/MEK initially occurs at its protein level, and not at its transcriptional level.

**Fig 3:**
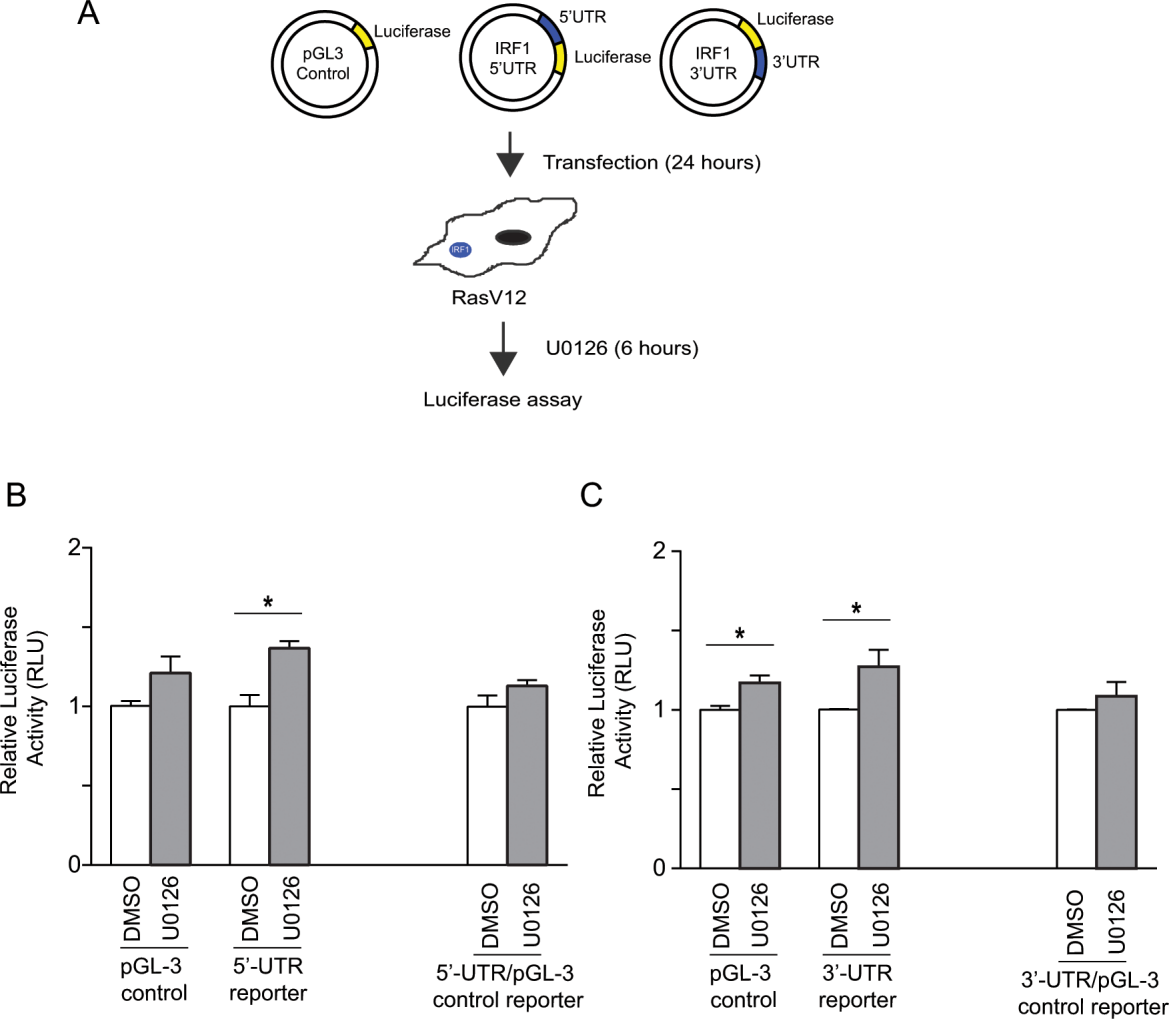
**Involvement of 5’ and 3’-UTR cis-acting elements in regulation of IRF1 translation by Ras/MEK** (A) Illustration of IRF1 3’- and 5’-UTR luciferase reporter constructs. Luciferase activities were measured in cell lysates obtained from RasV12 cells transfected with pGL3-control construct, (B) 5’-UTR or (C) 3’-UTR of IRF1 with or without U0126 treatment (20μM) for 6 hours. RLU were reported as compared with DMSO controls (n = 3, *P<0.05). Result shown is a representative of three independent experiments.

We next sought to study whether Ras/MEK activity modulates the translation of IRF1 mRNA. The *cis*-acting elements in 5’- and 3’-UTR of mRNAs are essential for the translational control of mRNA. To examine whether Ras/MEK regulates IRF1 translation, we constructed luciferase reporter constructs containing 5’- or 3’-UTR of IRF1 (Fig 3A). RasV12 cells were transfected with pGL3 control, IRF1 5’- or 3’-UTR reporter constructs, and then left untreated as a control or treated with U0126 for 6 hours. Translational activity of IRF1 5’-UTR construct was significantly increased in RasV12 cells treated with U0126 compared to those treated with DMSO (Fig 3B). However, since U0126 treatment also activated pGL-3 control construct in a non-specific manner, the promotion of IRF1 5’-UTR construct activity by MEK inhibition was not significant when compared to that of pGL-3 control construct by U0126. Similarly, U0126 treatment promoted the translational activity of both pGL-3 control and 3’-UTR reporter constructs (Fig 3C). Together, it is unlikely that Ras/MEK target the *cis*-acting elements in 5’- and 3’-UTR of IRF1 mRNAs to regulate its translation.

**Fig 4:**
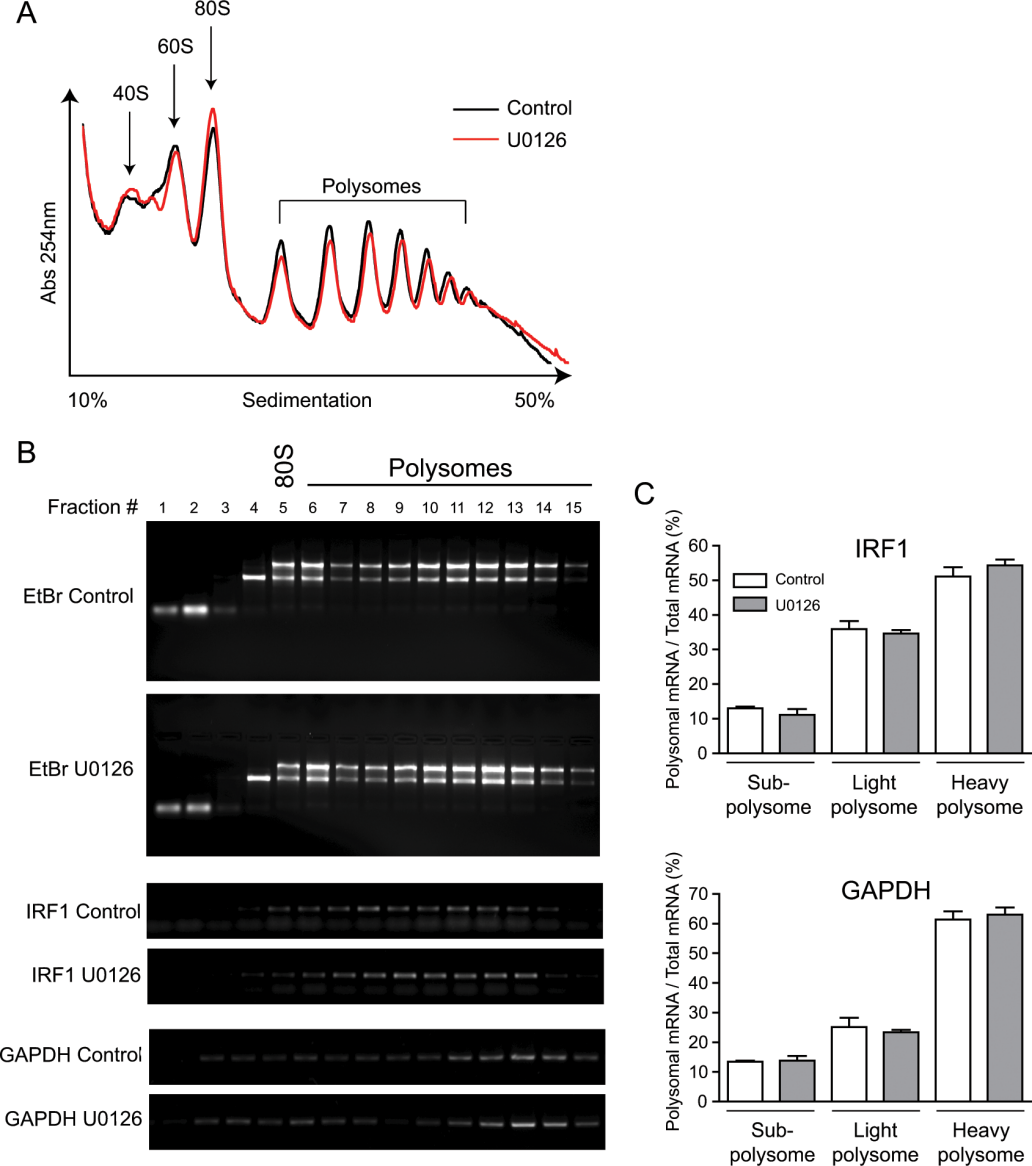
Translational control of IRF1 mRNA is independent from Ras/MEK activation. (A) Polysome profiles of RasV12 cells treated with or without U0126 (20 μM) for 2 hours were determined by recording the optical density (OD) of fractionated gradients at 254 nm. Peaks corresponding to 40S, 60S, 80S, and polysomes are indicated. (B) Ethidium bromide (EtBr) staining of RNA isolated from fraction #1 to #15 (Top panel). RT-PCR analysis of IRF1 (middle panel) and GAPDH (bottom panel) in each fractions. (C) RT-qPCR analysis of IRF1 and GAPDH in pooled fractions representing the sub-polysomes (fraction #1–5), the light polysomes (fraction #6–9), and the heavy polysomes (fraction #10–15). Data was represented as percentage of polysome-associated mRNA/total mRNA (n = 3, 3 independent experiments).

To directly determine whether Ras/MEK activation downregulates the translation of IRF1 mRNA, polysome analysis was conducted on RNA samples obtained from RasV12 cells treated with or without U0126 for 2 hours, as this was the time point when IRF1 protein level was significantly increased [1]. Analysis of polysome profiles revealed that the levels of 40S, 60S, 80S, and polysome complexes did not change upon U0126 treatment, indicating that the global mRNA translation was not affected by 2 hours of MEK inhibition (Fig 4A). To determine whether Ras/MEK regulates the polysome-loading of IRF1 mRNA, RNA was isolated from each fraction, converted into cDNA, and was analyzed by semi-quantitative RT-PCR for IRF1 and GAPDH (Fig 4B). Isolated RNA were examined on ethidium bromide gel to determine the fractions containing polysomes (top panel, fractions #6–15 contain polysomes). RT-PCR analysis of the ribosome-associated IRF1 mRNA between non-treatment control and U0126 treatment indicated that IRF1 mRNAs were equally distributed among the fractions #5–13 in the control group. In contrast, although IRF1 mRNA were observed in the same number of fractions, U0126 treatment slightly shifted a peak of ribosome-associated IRF1 mRNA to the fractions #9–13, indicating the possibility that MEK inhibition may promote translation of IRF1 mRNA. Polysome loadings of GAPDH mRNA, which were examined as a negative control, did not change by MEK inhibition. To further examine these results, the fractions representing sub-polysomes (fraction #1–5), light polysomes containing 2–5 ribosomes (fraction #6–9), and heavy polysomes containing 6 or more ribosomes (fraction #10–15) were pooled, and analyzed by quantitative RT-PCR for IRF1 and GAPDH (Fig 4C). The levels of IRF1 mRNA in these fractions did not change by U0126 treatment. Similarly, the polysome profile of GAPDH mRNA was not modulated by U0126 treatment. Based on these observations, it is unlikely that Ras/MEK controls IRF1 expression at the translational level.

## Materials and Methods

### Cells and reagents

NIH3T3 cells were obtained from the American Type Culture Collection (Manassas, VA, USA). RasV12 transformed NIH3T3 cells were generated as previously described [6]. IRF1deficient and wild type MEFs were established from Day 14 embryos of C57BL/6-Irf1^tm1Mak 47^ and C57BL/6J mice from the Jackson Laboratory (Bar Harbor, ME, USA), respectively. The animal care protocol (13-10-KH) was approved by the Institutional Animal Care Committee, in accordance with Canadian Council on Animal Care guidelines. All the animals were euthanized in a CO_2_ chamber before MEF isolation. All cell lines used in this study were maintained in high glucose Dulbecco's modified Eagle's medium (DMEM) (Invitrogen) with 10% fetal bovine serum (FBS) (GE Healthcare Life Sciences, Mississauga, ON, Canada). U0126 was purchased from Cell Signaling Technology (Danvers, MA). Antibodies to phospho-ERK-1/2 was purchased from Calbiochem, GAPDH (6C5) from Abcam (Toronto, ON, Canada), mouse IRF1 (M-20) and total ERK (sc-94) from Santa Cruz Biotechnology (Santa Cruz, CA, USA).

### Promoter and UTR reporter assay

For promoter reporter assay, promoter reporter constructs of GBP2, IRF1 variant 1&3, and IRF1 variant 2 were obtained by PCR amplification of mouse genomic DNA and ligation into the XhoI and HindIII sites of pGL3-Basic vector (Promega, Madison, WI). RasV12 cells (3 x 10^4^ cells/well), wildtype (4 x 10^4^ cells/well) or IRF1 deficient MEFs (4 x 10^4^ cells/well) were plated in 24-well plate and transfected with 1μg of the reporter plasmids using SuperFect Transfection Reagent (Qiagen). Twenty four hours after transfection, cells were treated with U0126 or IFN-α for additional 24 hours. Luciferase activity was measured by the Luciferase Assay System (Promega) and luminescence measured using Fluoroskan Ascent FL (Thermo Labsystems, Waltham, MA). For UTR reporter assay, 5’- or 3’-UTR sequence of IRF1 was PCR amplified using mouse pCMV-SPORT6-IRF1 (Thermo Fisher Scientific) as a template and subcloned into Xbal site or Ncol site in pGL3 Control vector. RasV12 cells were transfected with 0.5ug of reporter plasmids overnight and then treated with or without U0126 for 6 hours. The sequences of mouse IRF1 promoter variant 1, variant 2, variant 3, mouse IRF1 5’ UTR and 3’UTR are shown in S1 Fig.

### Polysome analysis

Polysome analysis was conducted as previously described [44]. RasV12 cells were cultured to approximately 80–90% confluency in 15-cm dish and treated with or without U0126 (20μM) for 2 hours. At the end of the U0126 treatment, cycloheximide was added to the culture media to a final concentration of 100μg/ml and incubated for 5 minutes to prevent ribosome runoff from mRNA. Cells were washed and scraped with ice-cold PBS supplemented with cycloheximide (100μg/ml), centrifuged, and lysed in hypotonic buffer (5mM Tris-HCl pH 7.5, 2.5mM MgCl_2_, 1.5mM KCl, complete protease inhibitor cocktail (Roche, Mississauga, ON, Canada)). The cell lysate was further supplemented with cycloheximide (100μg/ml), DTT (2mM), RNase inhibitor (100U), 10% Triton X-100 (final concentration of 0.5%), and 10% Sodium Deoxycholate (final concentration of 0.5%), centrifuged and then the supernatant was separated on a 10–50% sucrose gradient by centrifugation at 35,000 rpm for 2 hours. After separation, gradients were fractionated from the top of the gradient by pumping the chasing solution (60% w/v sucrose, 0.02% w/v bromophenol blue) from the bottom of the tube at 1.5 ml/min using an Isco density gradient fractionator (Teledyne Isco Inc., Lincoln, NE) and the RNA was monitored at 254 nm using the WinDaq data acquisition software (DATAQ Instruments, Akron, OH). RNA was isolated from each fraction, treated with DNase, and equal volume of RNA (5μl) from individual fractions was used for cDNA synthesis using the RevertAid H minus first strand cDNA synthesis kit (Thermo Fisher Scientific) according to the manufacture’s instruction. The levels of polysome associated IRF1 and GAPDH mRNA in each fraction was determined by semi-quantitative PCR using primers of IRF1 (5’ TCTTGCCCTCCTGAGTGAGT and 3’ TCTAGGGCCAGTGCTATGCT) and GAPDH (5’ GGGTGGAGCCAAACGGGTCA and 3’ GGAGTTGCTGTTGAAGTCGCA). For quantitative analysis, RNA isolated from fractions representing subpolysomes (fraction #2–6), early polysomes (Fraction #7–10), or heavy polysomes (fraction #11–15) were pooled, and measured by RT-qPCR.

### RT-qPCR

Quantitative RT-PCR (RT-qPCR) was performed in triplicate using the previously described validation strategies [8]. The primers used in the study are IRF1 (5’ CCTGGATTCCTGACTGTTGTCG and 3’ TGGCACATGCACAGCAAGAT) and GAPDH (5’ ATGTGTCCGTCGTGGATCTGA and 3’ TGCCTGCTTCACCACCTTCTT). RT-qPCR was conducted using SuperScript™ III Platinum SYBR Green One-Step RT-qPCR Kit with ROX (Life Technologies) and analyzed on the StepOnePlus qPCR system (Applied Biosystems, Foster City, CA, USA).

### Western Blot analysis

Cells were washed with PBS and lysed with RIPA buffer containing 0.1% SDS, 10 μg of aprotinin ml^-1^, 100 μg of PMSF ml^-1^ and 1% phosphatase inhibitor cocktail (Sigma). The samples were subjected to SDS-PAGE and transferred to nitrocellulose membranes (Bio-Rad). The membrane was blocked with 5% skim milk in TBS containing 0.1% Tween 20 and then incubated with the primary antibody, followed by secondary antibody (peroxidase-conjugated anti-rabbit, anti-goat or anti-mouse) (Santa Cruz Biotechnology). Specific bands were detected using Inmobilon Western (Milipore). Nuclear and cytoplasmic extracts were obtained using the Nuclear Extract Kit (Active Motif).

### Statistical analysis

One-way ANOVA with Tukey’s post-hoc test or Student’s *t* test was performed using GraphPad Prism 4.0c software (GraphPad Software, La Jolla, CA, USA).

## Discussion

In our previous study, we reported that MEK inhibition restores IRF1 expression in cells with Ras activation at both mRNA and protein levels [1]. In this study, to determine whether IRF1 downregulation by Ras/MEK occurs at the transcriptional or translational levels, we conducted IRF1 promoter assay in IRF1 deficient MEFs (Fig 2). We found that MEK inhibition does not increase IRF1 transcription in the absence of IRF1 protein, suggesting that Ras/MEK initially modulates IRF1 expression at its protein level such as translational regulation, posttranslational modifications or protein stability. As we reported previously, Ras/MEK does not regulate the stability of IRF1 protein [1]. Therefore, we further determined whether Ras/MEK activation downregulates IRF1 translation by conducting the UTR reporter analysis and polysome analysis.

To address whether Ras/MEK regulates the translation of IRF1 mRNA, we conducted 5’-UTR and 3’-UTR reporter assay as *cis*-elements on the UTRs of mRNAs are essential for translational regulation or micro RNA binding (Fig 3). The major problem we encountered in this experiment was the non-specific promotion of reporter activities by U0126 treatment. As MEK inhibition does not increase the rate of protein synthesis by cap-dependent translation [45], it is most likely that U0126 treatment increases the transcriptional activity of the pGL3 vector due to activation of its SV40 promoter. To address this, we also conducted the reporter analysis using a new set of UTR constructs with cytomegalovirus (CMV) promoter, but observed similar non-specific promotion of reporter activities by U0126 (data not shown). Therefore, an alternative future approach would be to use *in vitro*-transcribed RNA reporter constructs to eliminate the non-specific promotion. Nevertheless, these experiments demonstrate that Ras/MEK does not modulate translational efficiency of IRF1 mRNA via *cis*-elements on its 5’-UTR or 3’-UTR.

To further determine the involvement of Ras/MEK in IRF1 mRNA translation, we next conducted polysome analysis to determine whether MEK inhibition leads to the promotion of IRF1 mRNA translation. U0126 has been reported to substantially change the profile of polysome-associated mRNA in glial progenitor cell with activated Ras [46]. Unlike this study, we did not find any significant difference in the level of global translation in RasV12 cells in response to MEK inhibition (Fig 4A). It is likely due to differences in the cell types and the duration of U0126 treatment. Using polysome analysis, one of the IRF family members, IRF7, was previously found to be translationally modulated through regulation of eukaryotic translation initiation factor 4E (eIF4E) by its binding proteins (4E-BPs) [47]. As eIF4E is one of the downstream targets of the Ras/MEK pathway [48–51], we hypothesized that IRF1 is translationally regulated by eIF4E in a similar way to IRF7. However, it was not the case for IRF1 downregulation by Ras/MEK as ribosome binding to IRF1 mRNA was not modulated by U0126 treatment.

While IRF1 variant 1&3 promoters were activated either by MEK inhibition or IFN-α treatment, variant 2 promoter responded to U0126 treatment but not to IFN-α treatment (Fig 2A). The different responses of IRF1 variant 2 promoter to U0126 and IFN-α indicate that MEK inhibition and IFN-α stimulation activate IRF1 transcription via distinct mechanisms. Furthermore, these results suggest that IRF1 variant 2 may not play a role in IFN-α mediated antiviral defense. It is also possible that cytokines other than type I IFN activate its transcription. In summary, our study clearly demonstrates that IRF1 downregulation by activated Ras/MEK is independent from the translational control of IRF1 mRNA. As Ras/MEK activation does not regulate IRF1 stability either [1], it is most likely that posttranslational modifications of IRF1 is the mechanism underlying IRF1 downregulation by Ras/MEK, which we will investigate in future studies.

## Supporting Information

S1 Fig The sequences of mouse IRF1 promoter variant 1, variant 2 variant 3, mouse IRF1 5’UTR and mouse IRF1 3’UTR. (PDF)
