# Supplementary material for: IRF1 Downregulation by Ras/MEK Is Independent of Translational Control of IRF1 mRNA
Source: PLoS One. 2016 Aug 10;11(8):e0160529. doi: 10.1371/journal.pone.0160529 (PMC4980046; doi:10.1371/journal.pone.0160529)
Supplement: S1 Fig — (PDF) [file pone.0160529.s001.pdf]

Mouse IRF1 5’UTR

CCGCTGCGGGGCCTCTTGGTAGCCCAGAGTGGCCGTCGCGCGCACCCGCGAG
CAGCGCCGGGGGACTCGGATGTCCGCCTGCGCGCACTTTTGGCGGTCGCAGC
TTGGCTCCCAGCTCTTGCTTTCGGACGCGCAGTGTTCGGTGCAGAGGTTGGCT
CGCTGCCTTGACTGGCTGGACCGGGTCGTGAACTACTGGGCTTTCGGGAGGA
GGTGCCACAGCCACC

Mouse IRF1 3’ UTR

TTTGGGTCTCTGACCCGTTCTTGCCCTCCTGAGTGAGTTAGGCCTTGGCATCA
TGGTGGCTGTGATACAAAAAAAGCTAGACTCCTGTGGGCCCCTTGACACATG
GCAAAGCATAGTCCCACTGCAAACAGGGGACCATCCTCCTTGGGTCAGTGGG
CTCTCAGGGCTTAGGAGGCAGAGTCTGAGTTTTCTTGTGAGGTGAAGCTGGC
CCTGACTCCTAGGAAGATGGATTGGGGGGTCTGAGGTGTAAGGCAGAGGCCA
TGGACAGGAGTCATCTTCTAGCTTTTTAAAAGCCTTGTTGCATAGAGAGGGTC
TTATCGCTGGGCTGGCCCTGAGGGGAATAGACCAGCGCCCACAGAAGAGCAT
AGCACTGGCCCTAGAGCTGGCTCTGTACTAGGAGACAATTGCACTAAATGAG
TCCTATTCCCAAAGAACTGCTGCCCTTCCCAACCGAGCCCTGGGATGGTTCCC
AAGCCAGTGAAATGTGAAGGGAAAAAAAATGGGGTCCTGTGAAGGTTGGCT
CCCTTAGCCTCAGAGGGAATCTGCCTCACTACCTGCTCCAGCTGTGGGGCTCA
GGAAAAAAAAATGGCACTTTCTCTGTGGACTTTGCCACATTTCTGATCAGAG
GTGTACACTAACATTTCTCCCCAGTCTAGGCCTTTGCATTTATTTATATAGTGC
CTTGCCTGGTGCCTGCTGTCTCCTCAGGCCTTGGCAGTCCTCAGCAGGCCCAG
GGAAAAGGGGGGTTGTGAGCGCCTTGGCGTGACTCTTGACTATCTATTAGAA
ACGCCACCTAACTGCTAAATGGTGTTTGGTCATGTGGTGGACCTGTGTAAATA
TGTATATTTGTCTTTTTATAAAAATTTAAGTTGTTTACAAAAAAAAAAAAAAA

IRF1 promoter variant 1 and 3

AGGTGGAAATAACCTAAATGTCCTACAACAGGGTATTTATCTATCGGGTAAA
TTTTTATTTATCCTTATGACAGAAAACAGGTACCCATCACTAACAAGGCTGCA
TATGGACATTCCAGGACACAGGCTGACTTTCATTATGTATACCCACACAGTTA
TTTTGATGTAATTTGTAGTCTCTGGTTGCTTTCAGTAGGGTCTCTGTGGGCTTC
AAGAGTTACAAAGTTTCTCTCCCTAATCTCATTTTGAATTTTCCATTGGCTGGT
CAGAGTGTCTCCACAGAGCAAGTATACATCTGTTGCACAGAGAGCAGGTGTC
AGATTCAGAGTCATGTGATCAGAAGGCCCTGGTCTCTAGTGATTCTCTTAATT
TGCGACTTCAAATACCTCCGTATTTATTCTAGAGTCTCAATTCCTCCTTCCAAA
TCCTCCTGCCACCTTGAATCTCCTAGCTCGTCTATGAGGCAGTGACGTTACAG
GTACAATCGTCCATTGATGTGTGTATTACAGCTTGCAACATTCAAACTGGGTG
CTGTCCTAGACGGACAGCTAGGGTGAGGAAGGTGACACAAGAGAATGACAC
GGTAGAGGACTGATGTACTTGTGAGTCAGAAGCTGCGAGGAAGCGAATGAA
GAAGCCTGAGGGGCCAGAGTTCGGAGATGGAACATGTATACACTAGCCCAGC
CGCTAAGAGAAAGAACAAACTGGCAAGGTTTACTCGTGAACACCAGTTAACC
CGCTGCCCTTGCCATCCCCTTGGGACCTGGGTCCCCCCTTAGCCACAATTTCC
CTATGCTGCCCAGAACTTCTCCAGAGCCAGGCTTTGGGGACTCAGAAATGAC
TTCAAGACTATCAGAGAGGAACTCTCTCCCTAGGGCTACGTCGAGGATGGAG
AGCTGAAGTAGGTGTCATAGAAGGAACCACGGCCACTTCGGAATTTTATCCA
CTTCTTTGGTGTTAGGTAGTCCCAAGGAATAAGGGTCGCCTTGGTCACCGCTA

GCTAGAACCTCGTGTGGCACAGGAAAGCACACCCGCATCTTGTGCCCATGTA
AGCAAACACATCATCTCTCCCCATAAGGACAAGGTGTTCCCCCATCACTTCA
GACTAGCAAGTGAGCTATTGACATTTTATGGTTACGGAGTCCTTGAGTCCTTC
AACGGCTGGCCCTGCAGAGTCCAGGCTGGGAGTTCCTACTGCCCCGTATTTCC
AAGTTAGGCCTTCTGCCTCAATTTCCCAGAGCAGCCAAGTGGCAAGTAAGGC
GGTACAGGGACATAATCTCTCCCTTCTTGACACTTCATTACCAGACTGATTTT
GAGGGTGATTTAGTCCCGGGGACCCTTCTGGAGAGGGAATCAGGTGTTCTAG
AGACATAGGGAGTTAGGTGTCTCCCGTGGAGAAGGGGAAGACCATCATAGG
AGCCAGCAGGCTGGGAGTCTGCAGAAAGAGGGGGACGGTCTCGGCTTTCCAA
GACAGGCAAGGGGGCAGGGGAGTGGAGTGGAGCAAGGGGCGGGCCCGCGGT
AGCCCCGGGGCGGTGGCGCGGGCCCGAGGGGGTGGGGAGCACAGCTGCCTT
GTACTTCCCCTTCGCCGCTTAGCTCTACAACAGCCTGATTTCCCCGAAATGAT
GAGGCCGAGTGGGCCAATGGGCGCGCAGGAGCGGCGCGGCGGGGGCGTGGC
CGAGTCCGGGCCGGGGAATCCCGCT

IRF1 promoter variant 2
AGGTGGAAATAACCTAAATGTCCTACAACAGGGTATTTATCTATCGGGTAAA
TTTTTATTTATCCTTATGACAGAAAACAGGTACCCATCACTAACAAGGCTGCA
TATGGACATTCCAGGACACAGGCTGACTTTCATTATGTATACCCACACAGTTA
TTTTGATGTAATTTGTAGTCTCTGGTTGCTTTCAGTAGGGTCTCTGTGGGCTTC
AAGAGTTACAAAGTTTCTCTCCCTAATCTCATTTTGAATTTTCCATTGGCTGGT
CAGAGTGTCTCCACAGAGCAAGTATACATCTGTTGCACAGAGAGCAGGTGTC
AGATTCAGAGTCATGTGATCAGAAGGCCCTGGTCTCTAGTGATTCTCTTAATT
TGCGACTTCAAATACCTCCGTATTTATTCTAGAGTCTCAATTCCTCCTTCCAAA
TCCTCCTGCCACCTTGAATCTCCTAGCTCGTCTATGAGGCAGTGACGTTACAG
GTACAATCGTCCATTGATGTGTGTATTACAGCTTGCAACATTCAAACTGGGTG
CTGTCCTAGACGGACAGCTAGGGTGAGGAAGGTGACACAAGAGAATGACAC
GGTAGAGGACTGATGTACTTGTGAGTCAGAAGCTGCGAGGAAGCGAATGAA
GAAGCCTGAGGGGCCAGAGTTCGGAGATGGAACATGTATACACTAGCCCAGC
CGCTAAGAGAAAGAACAAACTGGCAAGGTTTACTCGTGAACACCAGTTAACC
CGCTGCCCTTGCCATCCCCTTGGGACCTGGGTCCCCCCTTAGCCACAATTTCC
CTATGCTGCCCAGAACTTCTCCAGAGCCAGGCTTTGGGGACTCAGAAATGAC
TTCAAGACTATCAGAGAGGAACTCTCTCCCTAGGGCTACGTCGAGGATGGAG
AGCTGAAGTAGGTGTCATAGAAGGAACCACGGCCACTTCGGAATTTTATCCA
CTTCTTTGGTGTTAGGTAGTCCCAAGGAATAAGGGTCGCCTTGGTCACCGCTA
GCTAGAACCTCGTGTGGCACAGGAAAGCACACCCGCATCTTGTGCCCATGTA
AGCAAACACATCATCTCTCCCCATAAGGACAAGGTGTTCCCCCATCACTTCA
GACTAGCAAGTGAGCTATTGACATTTTATGGTTACGGAGTCCTTGAGTCCTTC
AACGGCTGGCCCTGCAGAGTCCAGGCTGGGAGTTCCTACTGCCCCGTATTTCC
AAGTTAGGCCTTCTGCCTCAATTTCCCAGAGCAGCCAAGTGGCAAGTAAGGC
GGTACAGGGACATAATCTCTCCCTTCTTGACACTTCATTACCAGACTGATTTT
GAGGGTGATTTAGTCCCGGGGACCCTTCTGGAGAGGGAATCAGGTGTTCTAG
AGACATAGGGAGTTAGGTGTCTCCCGTGGAGAAGGGGAAGACCATCATAGG
AGCCAGCAGGCTGGGAGTCTGCAGAAAGAGGGGGACGGTCTCGGCTTTCCAA
GACAGGCAAGGGGGCAGGGGAGTGGAGTGGAGCAAGGGGCGGGCCCGCGGT
AGCCCCGGGGCGGTGGCGCGGGCCCGAGGGGGTGGGGAGCACAGCTGCCTT

GTACTTCCCCTTCGCCGCTTAGCTCTACAACAGCCTGATTTCCCCGAAATGAT
GAGGCCGAGTGGGCCAATGGGCGCGCAGGAGCGGCGCGGCGGGGGCGTGGC
CGAGTCCGGGCCGGGGAATCCCGCTAAGTGTTTAGATTTCTTCGCGGCGCCG
CGGACTCGCCAGTGCGCACCACTCCTTCGTCGAGGTAGGACGTGCTTTCACA
GTCTAAGCCGAACCGAACCGAACCGAACCGAACCGAACCGGGCCGAGTTGC
GCCGAGGTCAGCCGAGGTGGCCAGAGGGACCCCAGCATCTCGGGCATCTTTC
GCTTCGTGCGCGCATCGCGTACCTACACCGCAACTCCGTGCCTCGCTCTCCGG
CACCCTCTGCGAATCGCTCCTGCAGCAAAGGTGAGCTCGCCGGAGCTGCGCG
GGCACCGACGGGTGCCGTGGAACAAAAGGCGCACGGGACACCAGGAAGTGG
GGGCCAGAGACACCCTGGGTCGCGGTGGCGCGCTGGCTCGGGAGGGCGCTG
CAAGCGGGCGGGAGGCGGGCCGCGGCGTGCACCGCCCTCTGGCCGCGCTCG
GCGGTCTGCGTGCGCGGAG

S1 Fig. The sequences of mouse IRF1 promoter variant 1, variant 2, variant 3, mouse IRF1 5’ UTR and mouse IRF1 3’UTR.
